# Geographical range overlap networks and the macroecology of species co-occurrence

**DOI:** 10.1371/journal.pone.0266275

**Published:** 2022-04-06

**Authors:** Marcio R. Pie, Fernanda S. Caron

**Affiliations:** 1 Biology Department, Edge Hill University, Ormskirk, Lancashire, United Kingdom; 2 Departamento de Zoologia, Universidade Federal do Paraná, Curitiba, Paraná, Brazil; Southeastern Louisiana University, UNITED STATES

## Abstract

Direct interactions among species are only possible if there is some overlap in their geographical distributions. However, despite intense focus of macroecological research on species geographical ranges, relatively little theoretical and empirical work has been done on the evolution of range overlap. In this study we explore a simple model of range overlap based on a log-normal distribution of species range sizes along a one-dimensional domain, with or without absorbing boundary conditions. In particular, we focus on the mean and variance of range overlap distributions, as well as the topology of the resulting overlap networks with respect to their degree distribution, evenness, and betweenness scores. According to the model, there is an approximately linear relationship between many aspects of the distribution of range overlaps and their underlying species distributions, such as their mean and variance. However, the expected mean number of non-zero range overlaps for a given species varied from linear to convex depending on the variance of the underlying geographical range distribution. The expected topology of range overlap networks varied substantially depending on the mean and variance in the corresponding geographical distributions, particularly in the case of the degree and closeness distributions. Finally, we test the expectations of our model against five datasets of altitudinal distributions of Neotropical birds. We found strong departures from the expectations based on our model, which could potentially result from phylogenetic niche conservatism related to altitudinal gradients in environmental conditions, or from the asymmetric colonization of mountains by species from lowlands. Potential applications of range overlap networks to a variety of ecological and evolutionary phenomena are discussed.

## Introduction

Species range sizes have been a major focus of macroecological research since the early days of the discipline [1–3]. In particular, studies on a variety of organisms and habitats indicate that the distribution of range-sizes commonly is approximately log-normal, with a left-skew, regardless of taxon or biogeographical region [3–8]. Ultimately, there are three basic mechanisms that drive range size evolution: speciation, extinction, and transformation (i.e. the temporal dynamics of the range sizes of species during their lifetimes) [9], but the understanding of how those mechanisms actually translate into the shapes of range size distributions that one might find empirically is still limited. Despite such uncertainty regarding the processes underlying species range size distributions, the discovery of such widespread near-log-normal distributions provides the foundation for many other areas within macroecology and biogeography. For instance, the fact that the ranges of most species tend to be small has important implications for the discovery of new species [4, 10], as well as their conservation (e.g. [11, 12]).

Given the strong interest in range-size distributions, it is perhaps surprising that little empirical and theoretical work has been done on patterns of range overlap. Important advances have been achieved regarding the shift to sympatry in closely-related species [13–15], yet many important aspects of range overlaps are largely overlooked. For instance, to the best of our knowledge, no study to date has investigated the shape of the distribution of range overlaps for any taxon. In particular, one might expect that a set of species with large average range size within a given region would be more likely to overlap its range with other species. A deeper understanding of the drivers of range overlaps might provide valuable insight into a variety of long-standing ecological and evolutionary questions, such as the importance of biotic interactions in determining range limits [7], and the feasibility of joint species distribution models [16–18].

A particularly interesting approach to the study of geographical distributions was pioneered by Araújo et al. [19] and consists of describing the patterns of species range overlaps in the form of networks. Using data on European amphibians, reptiles, birds, and mammals, they showed that range overlap networks display properties shared with other complex networks, namely that most species are poorly connected to other species in the network and only a few are highly connected. In addition, they suggested that poorly connected species tended to be those more susceptible to climate change [19]. Their study provided a substantial shift from traditional studies on species co-occurrence at local scales, particularly those testing community assembly rules (e.g. [20]). However, such an approach is still poorly explored, possibly because it is still difficult to determine which aspects of range overlap networks are expected or unusual without predictions based on null models.

In this study we explore a few general, but still poorly understood aspects of the evolution of range size overlap. First, we describe a simple model of range overlap based on a log-normal distribution of species range sizes along a one-dimensional domain with and without absorbing boundary conditions. Despite its simplicity, we demonstrate that this model can generate several interesting predictions regarding the distribution of range-size overlaps, such as the mean and variance of range overlap distributions according to the underlying mean and variance of range sizes. Second, we explore matrices of pairwise range overlaps among a set of species that can be represented as range overlap networks (i.e. co-occurrence networks *sensu* [19]). Finally, we compare the predictions from the model with a dataset of altitudinal distributions of avian species from different regions of the Neotropics.

## Materials and methods

### A null model of range overlap

**Fig 1:**
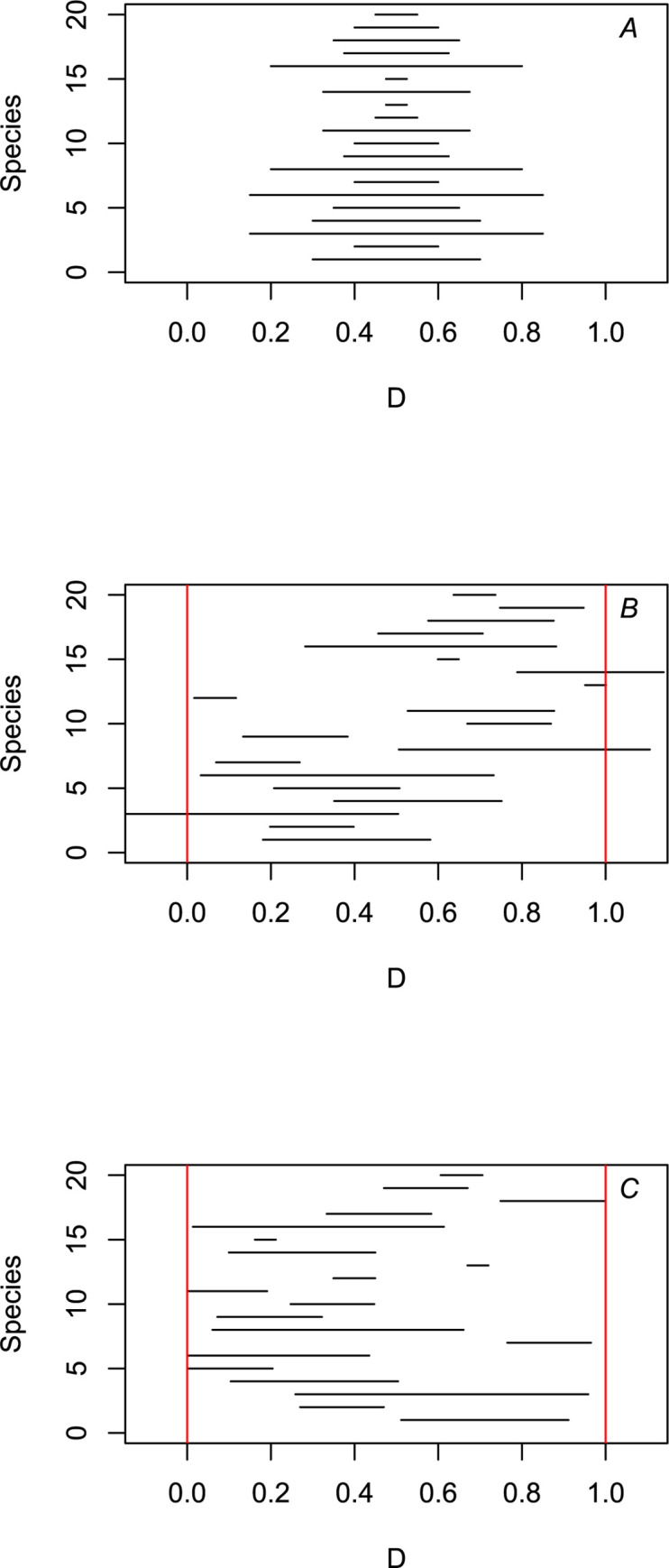
The main steps in generating the patterns of range overlap in our model. Diagram illustrating: (A) the range size of each species was simulated according to a log-normal distribution; (B) the midpoint of each range was uniformly distributed along the available space as ~U(0,*D*), in which *D* is the size of the entire available domain; and (C) ranges are truncated if they exceeded *D* so that all ranges fit within the available domain. See text for details.

We simulated ranges of *S* species along a single continuous dimension in which *D* is the size of the entire domain. (To facilitate comparisons, here we will set *D* to 1 in all simulations.) The range size of each species was simulated according to a log-normal distribution (Fig 1A). For instance, a simulation with a mean of -0.5 on a log scale leads to a mean range size of ~1 size unit on a linear scale. The midpoint of each range was uniformly distributed along the available space as ~*U*(0,*D*) (Fig 1B) and then truncated if it exceeded *D* so that all ranges fit within the available domain (Fig 1C). This truncation was designed to reflect the possibility that a species could potentially be distributed beyond the limits of *D*, but was prevented from doing so due to geometric constraints in the available geographical space. We defined ρ as the ratio between the average species range size (prior to truncation) with respect to the size of the entire domain *D*. The size of the range overlap between a pair of species was defined as the intersection along *D* in which both species are co-distributed. The obtained results were compared with similar simulations in which no truncation was performed. To facilitate visualization, range sizes, their overlaps, and *D* were always on a natural logarithmic scale. The general approach of the model presented here follows a long tradition of similar models in ecology, such as the broken-stick model [21], and the mid-domain effect [22, 23].

The main aspects of this model that were compared across simulations were the range size distribution, the range overlap size distribution, and the average number of overlapping species (i.e. for each species, how many other species showed nonzero range overlap. This also corresponds to the average node degree in the context of an overlap network—see below). In particular, we assessed (1) the relationship between the mean and variance in range size distributions and the average number of overlapping species (also known as linkage density in network analyses; [24]); (2) the relationship between mean and variance in range size distribution in the resulting range overlap distribution’s mean and variance; and (3) the topological properties of the resulting range overlap network. In the latter, we computed an incidence matrix based on whether each pair of species overlapped and then generated the corresponding unipartite graph. For the simulations in (1), (2), and (3), we generated 10,000, 1,000, and 200 species ranges, respectively. We chose relatively large numbers in relation to typical ecological datasets to allow for more precise visualization of the expected results of the model. Although our simulations are useful to provide qualitative predictions for the relationships among the studied variables, more rigorous tests of empirical data should require taking into consideration departures from log-normality and the stochastic effects of the more limited sample sizes available in biogeographical studies. For all simulations, we varied the standard deviation and mean of the underlying range size distribution to assess how the relationship between the variables would be affected. More specifically, we varied the standard deviation from 0 to 10 or maintained the range within these limits, and the mean from –4 to 0.5. These values correspond to parameters for the log-normal distribution, so in a linear scale, these values range approximately from 0.018 to 1.649 for the mean, which indicates that the ranges are on average 1.8% to 164.9% of the size of the domain (*D* = 1 in this study).

Three network metrics were explored in the range overlap network and calculated using the sna 2.6 package [25]: the degree distribution (the distribution of the number of overlapping species across the network, in which the more individuals a particular species overlaps with, the greater its degree score), closeness (the extent to which a node is close to all other nodes in a graph, where a high closeness indicate species with more direct overlaps, whereas a lower closeness score means that a species is connected to other species but with a higher frequency of indirect paths through other species’ overlaps), and betweenness (the frequency with which a node falls between pairs of other nodes on the shortest or geodesic paths connecting them, in which a species with a high betweenness score may connect several different species, being able to link disparate parts of the network—see [26] for more information on these network metrics). For a detailed review of networks of species interactions and the most used metrics see [27].

### Empirical analyses

We tested the predictions of our model against empirical data using the altitudinal distribution of neotropical birds available in [28] for five different locations. Although our model was developed in the context of geographical ranges, we believe that altitudinal distributions can be a good first approximation for empirical tests, considering that these two types of distribution can be analogous. In addition, altitudinal distributions vary in only one dimension, which is a good first approximation to understand their geographical distributions. The dataset used here [28] is the result of years of compilation of field works and several other published and unpublished sources on the ecological and distributional data of birds. The authors of this compilation pointed out that there may be errors in the dataset, as it has data on over 4,000 species, but they attempted to double-check to the extent possible. Particularly, it is important to highlight that because of the nature of the data, the elevational information may not represent the real elevational distribution of a species, as the lowest and highest boundaries may not happen in the same mountain range, that is, the minimum and maximum boundaries are across the elevational distribution of the entire region. The selected locations were Madrian Highlands (MAH) (N = 384 species), Chiriquí-Darién Highlands (CDH) (N = 278 species), Central Andes (CAN) (N = 801 species), Northern Andes (NAN) (N = 768 species), and Tepuis (TEP) (N = 142 species). We excluded from the analyses those species that had an altitudinal range equal to 0 (i.e. species with only a single altitudinal record). In each dataset, we defined the size of *D* as its respective difference between uppermost and lowermost altitudinal records. We used the empirical range sizes to fit a log-normal distribution using the "fitdistr" function in the MASS 7.3–51.4 package [29]. We performed simulations (n = 100) of the expected range overlap according to the estimated mean and standard deviation of altitudinal range size distributions and compared them with the empirical data. We also calculated the degree distribution, betweenness, and closeness of half of the simulated networks, using the sna 2.6 package [25], and contrasted them with the empirical data for each location. All analyses and simulations were carried out in R 3.6.0 [30].

## Results

### Null model of range overlap

**Fig 2:**
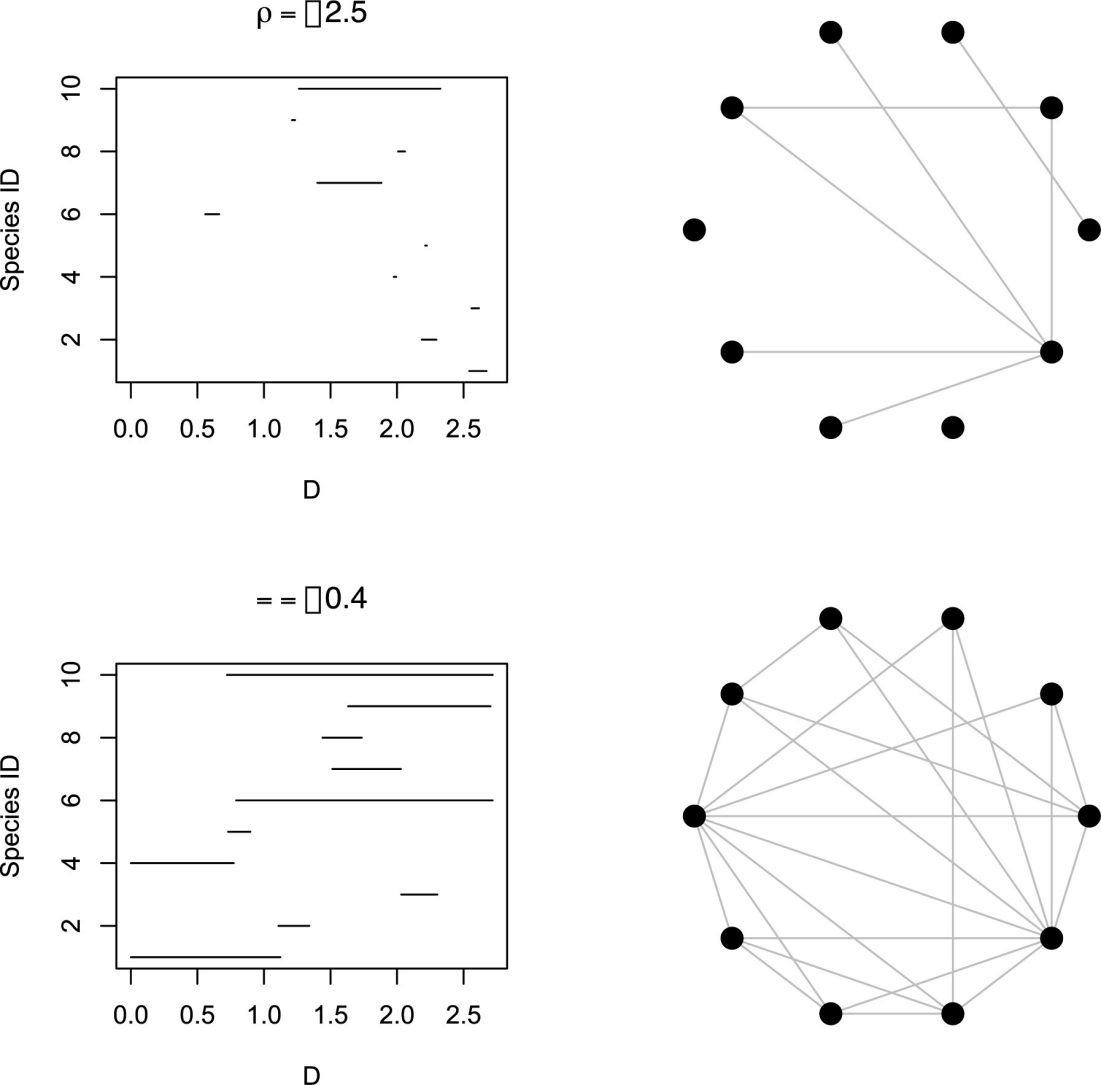
How variation in ρ would affect the pattern of range overlap and the resulting overlap network. ρ corresponds to the ratio between mean range size and the size of the entire domain *D*. The first row represents a situation in which the average range size is small with respect to the domain *D*, leading to little overlap and a sparse overlap network, whereas the second row reflects how a larger ρ would not necessarily make all species become distributed across *D*, yet the resulting overlap network shows considerable connectance.

Before exploring the properties of the model, it is useful to first visualize examples of how variation in ρ would affect both the pattern of range overlap and the resulting overlap network (Fig 2). When ρ = -2.5 (0.082 in linear scale), there is little overlap between ranges and the overlap network is sparse. However, in the case of ρ = -0.4 (0.670 in linear scale), most species encompass relatively little of *D* and yet the resulting overlap network is fairly dense, suggesting that the pattern of range overlap in a set of species might not be immediately intuitive given the knowledge of data on species ranges.

**Fig 3:**
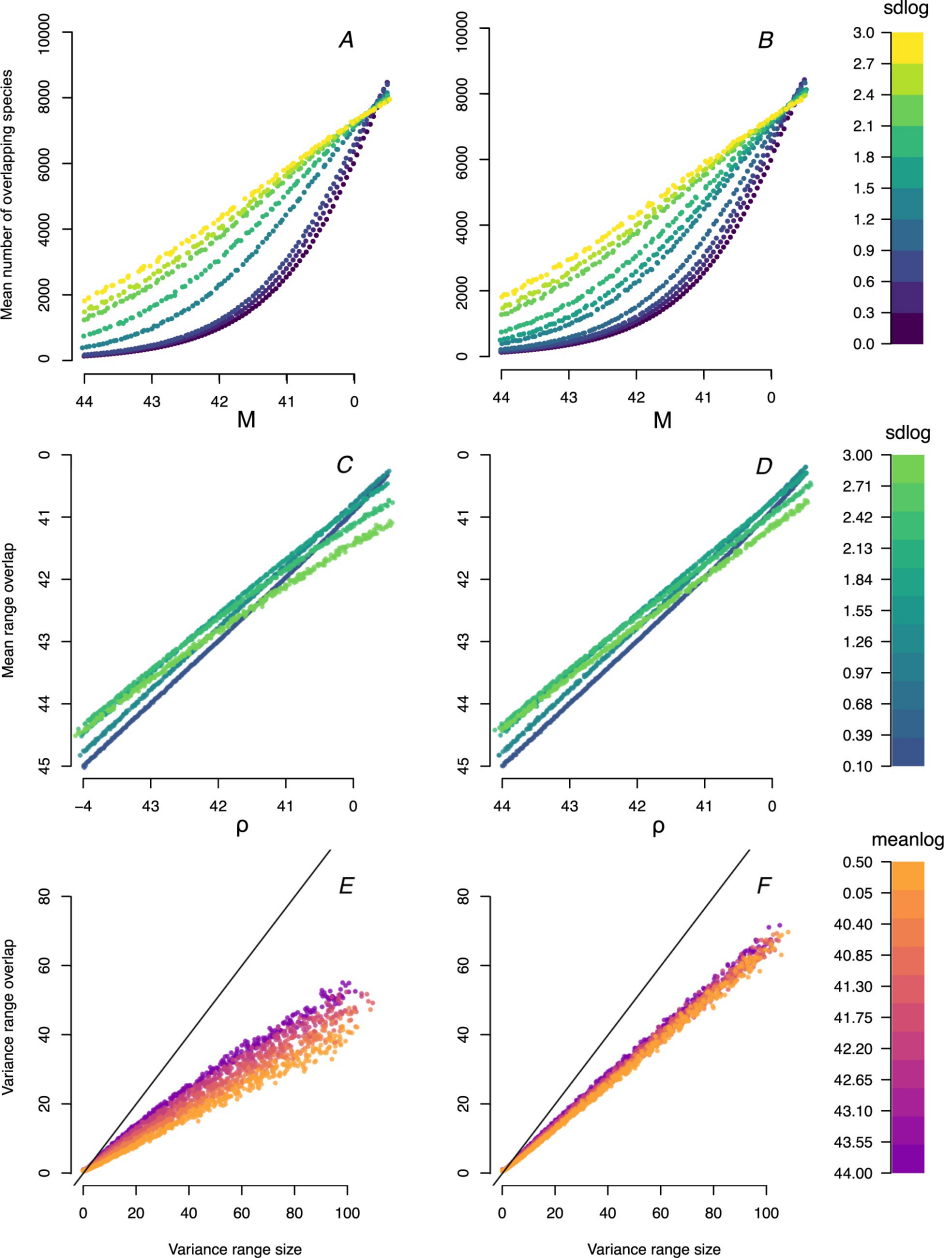
Simulations of our model according to variation in parameter space. Results with truncation are presented in the left column and without truncation in the right column. Each point represents a simulated dataset with 10,000 species in the first row and 1,000 species in the second and third row. (A, B) relationship between the mean number of overlapping species and ρ, with colors representing differences in the standard deviation of the underlying range size distributions (sdlog); (C, D) relationship between mean range overlap and ρ, with colors representing differences in the standard deviation of the underlying range size distributions (sdlog); (E, F) relationship between variance in range size and the range overlap distributions, with colors representing differences in the mean of the underlying range size distributions (meanlog).

The results of simulations of datasets with 10,000 and 1,000 species, with and without truncation, are shown in Fig 3. As expected, the mean number of overlapping species increases with their mean range size (ρ; Fig 3A and 3B). However, the shape of this relationship was directly affected by the variance in the underlying range size distribution (sdlog in Fig 3). When their variance (sdlog) is small, the relationship between the mean number of overlapping species and ρ is convex, becoming increasingly linear as the variance in range sizes becomes larger (Fig 3A and 3B). On the other hand, there was a positive relationship between ρ and mean range overlap throughout most of the parameter space, only becoming slightly concave in high values of ρ and when the variance in the underlying range size distribution (sdlog) was high (Fig 3C and 3D). Interestingly, the relationship between the variance in range size and variance in range overlap was linear, with modest decreases in slope with increasing range size (meanlog; Fig 3E and 3F). The results of truncated (Fig 3A, 3C, and 3E) and non-truncated (Fig 3B, 3D, and 3F) were qualitatively very similar, indicating that the predictions of our model are robust to the possibility of species potentially being able to be distributed farther than the limits of *D*.

**Fig 4:**
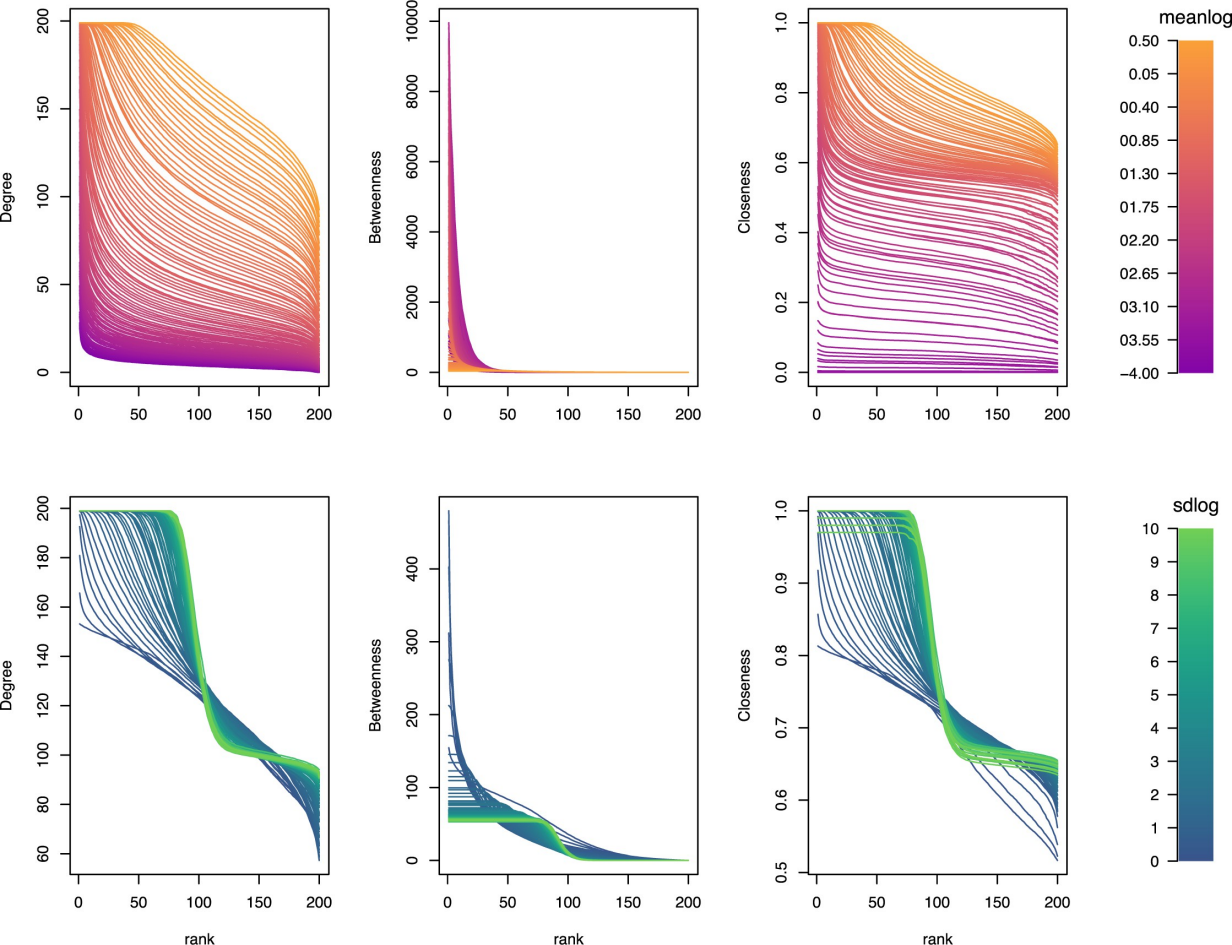
Variation across nodes in simulated range overlap networks (without truncation), namely degree distribution, betweenness, and closeness. Top Row: simulations in which the standard deviation in the range size distribution was set to 1 on a log scale, while the mean of the range size distribution (meanlog) was varied systematically; Bottom Row: simulations in which the mean of the range size distribution was set to 0 on a log scale, while the standard deviation of the range size distribution (sdlog) varied systematically.

The topological properties of overlap networks varied considerably with respect to the mean and variance of the underlying range size distribution (Fig 4). For instance, for non-truncated simulations, the distribution of node values for the degree distribution and closeness changed from more homogeneous to asymmetric as range size increased (meanlog in Fig 4), whereas the opposite was found for betweenness (Fig 4, first row). On the other hand, changing the variance in the range size distribution (sdlog; while maintaining the mean range size fixed to 0 on a log scale) had a complex influence on network metrics, with high variance levels leading to the formation of plateaus with many species with similar scores (Fig 4, second row). Results from truncated simulations were nearly indistinguishable from non-truncated simulations (S1 Fig).

### Empirical results

**Fig 5:**
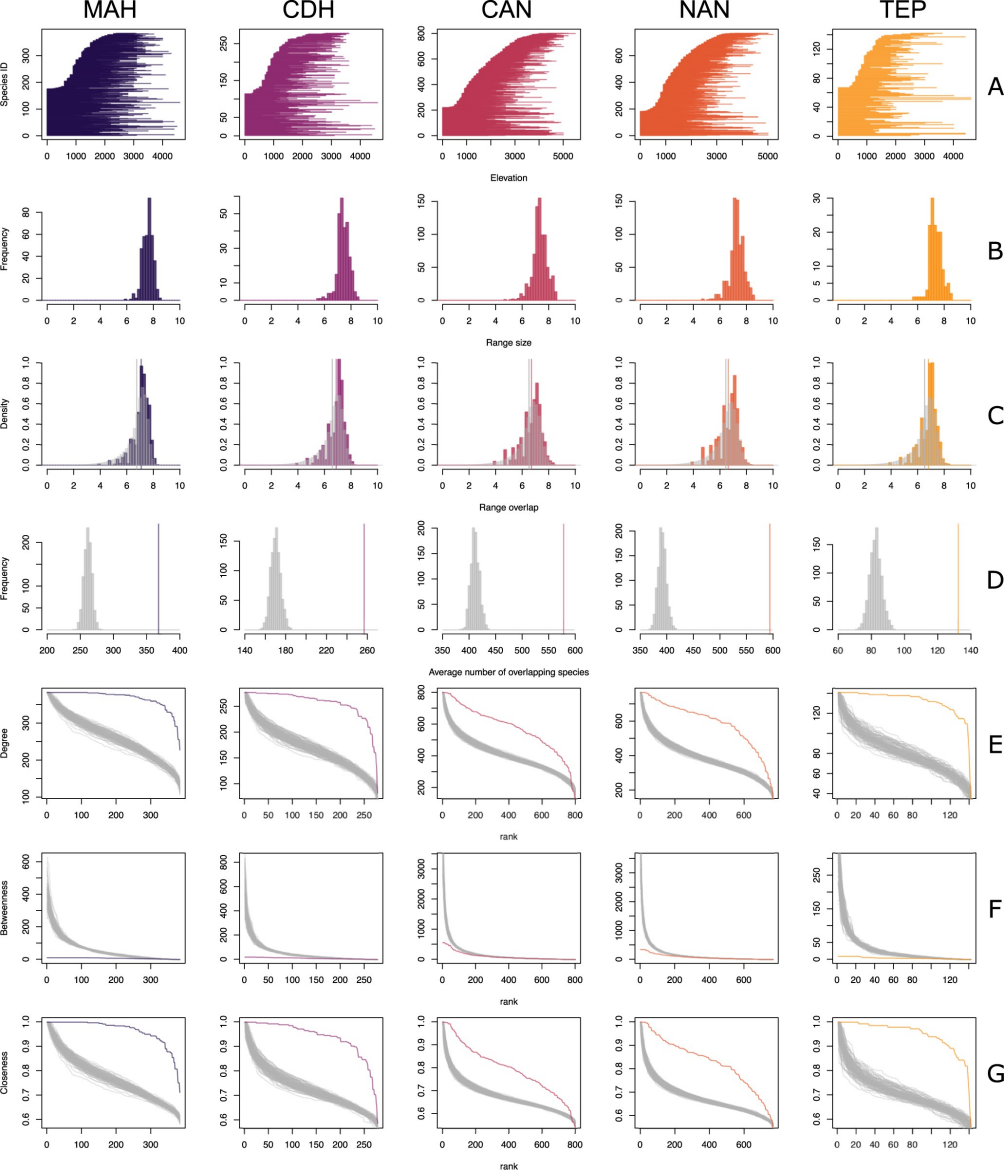
Comparison between empirical data on the altitudinal distribution of Neotropical birds and the expectations based on our model. The results showed are of simulations without truncation. Locations are: Madrian Highlands (MAH) (N = 384 species), Chiriquí-Darién Highlands (CDH) (N = 278 species), Central Andes (CAN) (N = 801 species), Northern Andes (NAN) (N = 768 species), and Tepuis (TEP) (N = 142 species): (A) altitudinal distribution of the species in each location, ranked according to the lowest limit to facilitate visualization; (B) altitudinal range size distribution; (C) altitudinal range overlap distribution (gray histogram indicates the expectation of the range overlap distribution based on 100 model simulations using mean and standard deviation calculated directly from the empirical data, and the colored and gray vertical lines indicate the observed and simulated mean range overlap, respectively); (D) distribution of the simulated number (n = 100) of overlapping species (histogram) and the observed means for each location (vertical lines); (E) degree distribution across nodes in the empirical datasets (colored lines) and expectations based on simulations (n = 50) (gray lines); (F) betweenness across nodes in the empirical datasets (colored lines) and expectations based on simulations (n = 50) (gray lines); (G) closeness across nodes in the empirical datasets (colored lines) and expectations based on simulations (n = 50) (gray lines).

A broad comparison between the predictions of our model and the empirical data on avian altitudinal distributions is shown in Fig 5. Although they involve different geographical regions and species compositions, the altitudinal distributions across all five regions were qualitatively similar. In particular, one pattern that becomes immediately obvious in all datasets is that many more species are constrained in their distribution by the lowest than by the highest altitudinal limit (Fig 5A), which is an aspect of altitudinal distributions which might not be properly accounted for by our model. The distribution of range sizes and range overlaps shared similar distributions and shapes (Fig 5B and 5C). The mean of the range overlaps is slightly smaller than the underlying range sizes, but the overall shape of the range overlap distribution was well predicted by our model. Interestingly, there were obvious departures from our model: empirical datasets had more range overlapping species (Fig 5D), higher degree and closeness scores (Fig 5E and 5G), and lower betweenness (Fig 5F) than expected. In addition, the distribution of the simulated overlap sizes (gray bars on Fig 5C) was very close to the observed data. The results from truncated simulations were very similar to non-truncated simulations (S2 Fig).

## Discussion

One could argue that the publication of Diamond [31] single-handedly gave rise to the modern study of species co-occurrences in ecological communities. In particular, the distribution of different bird species across the Malay Archipelago was interpreted as strong evidence for competition driving community assembly by leading to "checkerboard" distributions across different islands [31]. The dispute over the validity of Diamond’s assembly rules spurred the development of a smorgasbord of null models of species co-occurrence [20, 32–34]. It is difficult to overestimate how prolific this approach has been over the past four decades, which is still providing valuable insight into how positive or negative interactions might determine community composition [35, 36] and to inform joint species distribution modeling [17, 18]. However, the focus of the study of species co-occurrence has been essentially at the level of local community composition and its consequences for biotic interactions, whereas the actual distribution of the species in the regional pool has been essentially taken as a given (but see [37]). In this study we provide a complementary, top-down approach that begins by focusing on the level of entire geographical distributions. In particular, we sought to predict properties of the distribution of range overlaps from the underlying distribution of range sizes. We showed that, as the mean range size increased with respect to the available domain, there was a corresponding, linear increase in mean range overlap, which became slightly concave when the mean and variance in the range size were high (Fig 3C and 3D). In addition, the mean number of overlapping species showed a concave relationship to range size when its variance was low, which became increasingly linear as the variance in range size became larger (Fig 3A and 3B). We recognize that our model is a first approximation and that we have not incorporated several important mechanisms, such as the influence of biotic interactions or constraints in range position due to phylogenetic niche conservatism, yet we believe that the approach presented here can provide an important step in that direction.

Our study also expanded on the idea of Araújo et al. [19] of species co‐occurrence networks. (Given that the idea of co-occurrence has been linked in the literature to mechanisms at the local level, we chose to use the term "overlap networks" instead to avoid confusion.) In particular, we showed that variation in the mean and variance of range size distributions had profound effects on the resulting overlap networks. For instance, larger range sizes were associated with more asymmetry across nodes in the distributions of degree and closeness scores (i.e. if average range size is large, species with more connections tend to be disproportionately more highly connected than those in which average range size is smaller), whereas betweenness scores showed the opposite effect (i.e. if average range size is large, species tend to show more similar values of betweenness; Fig 4). This unintuitive complexity in the relationship between range size distributions and the resulting range overlap is intriguing and might have important consequences for a variety of ecological and evolutionary phenomena. Many models of equilibrial diversification dynamics typically assume a single, regional "carrying capacity" that determines the number of species that a region can support (e.g. [38]). However, that approach assumes a mean field situation in which all species equally compete for resources, which would be analogous to completely connected range overlap networks, which are very unlikely in practice. Explicitly incorporating range overlap networks might provide valuable insight into more realistic models of macroevolutionary dynamics and its consequences for phenotypic evolution in the case of both positive or negative coevolutionary interactions. Second, overlap networks seem particularly useful to understand probabilities of host shifts in host-parasite interactions. In particular, host species with highly connected range overlap networks might serve as hubs with a high probability of host shifts [39, 40]. Finally, range overlap networks might facilitate the study of the relationship between species coexistence and phenotypic evolution by providing a means to directly assess which species are more likely to experience interspecific biotic interactions [41, 42].

There were some intriguing differences between the empirical data on avian altitudinal distributions and the predictions in our model. In general, for all regions, species showed higher frequency and size of range overlaps, also resulting in different network metrics (Fig 5). This may reflect the fact that the mountain ranges of the data used are the range over the entire location, not in a single mountain, so there can be overlaps between species that in reality do not share the same mountain. On the other hand, if we assume that the data reflect the accurate elevational distribution of species, there are two alternative (non-exclusive) mechanisms to explain these departures. First, closely-related species might share similar environmental preferences (phylogenetic niche conservatism), leading their distributions to be more similar than expected by our model. This effect might be explored further by assessing the degree of range overlap and the corresponding average phylogenetic distance between species. Alternatively, as indicated above, species altitudinal ranges are considerably more likely to reach the lower altitudinal limit than the upper altitudinal limit. Given that there are lowland species that extend their ranges upward, this asymmetric source of species to montane communities might lead to this asymmetry, leading to higher range overlap than expected by our model. Regardless of the underlying mechanisms, our results are not consistent with negative (e.g. competitive) interactions limiting altitudinal range overlaps within each region. Although these results are intriguing, they are simply used to illustrate our approach, as the sets of species of each region might not necessarily be representative of the communities within a single mountain.

It is important to emphasize that there are some important caveats to the interpretation of range overlap networks. First, the existence of range overlap between a pair of species does not necessarily mean that they actually interact ecologically [43]. For instance, species with overlapping ranges might occupy different microhabitats or even be active at different times of the day. In this sense, range overlap networks can be envisioned as indicating the potential for ecological interactions, in the sense of being a necessary but not sufficient condition for direct interactions to occur. Second, we chose as a first approximation a one-dimensional model of range size, yet geographical ranges are inherently bidimensional, particularly in terrestrial organisms. One of the main challenges to extending our framework to two dimensions would be the choice for the shape of the geographical distributions (e.g. circles or squares with latitudinal and longitudinal limits), but these limitations should be relatively straightforward to address. Finally, our approach here is both binary (i.e. either species overlap their ranges or not) and static, yet both of those limitations are valuable opportunities for future extensions of our approach. For instance, one could include information on the extent of range overlap between species when computing their overlap networks so that they become more quantitative. However, one should keep in mind that range overlap is almost invariably asymmetrical, such that the area of overlap between any pair of species is likely to represent a different percentage of their entire range. Also, geographical ranges are dynamic, particularly over evolutionary time [7]. One interesting way to incorporate a temporal dimension into overlap networks would be to weigh edges based on the phylogenetic distance between species pairs. This could reflect that a pair of congener species are more likely to interact than two species from different families.

To the best of our knowledge, our study represents the first attempt to explicitly predict range overlap distributions from their underlying range size distributions, and many of our results seem particularly amenable to analytical solutions. As network theory has been increasingly applied to ecological phenomena, a large variety of potential metrics have been proposed and can be used to reveal different aspects of network organization (see [27] for a recent review). In this study we chose to focus on three commonly used metrics that should serve as a first approximation to the characterization of range overlap networks. However, that does not mean that other metrics, such as network connectance and intervality [27], will not lead to important insight. On the other hand, one must refrain from the temptation of estimating as many metrics as possible simply because they can be calculated. Rather, metrics chosen should be those most meaningful in the context of range overlap networks. A particularly promising area would be the integration of different range overlap networks, such as the construction of bipartite networks for sets of interacting species (e.g. host-parasite or plant-pollinator networks). In particular, the comparison of actual ecological interaction networks and range overlap networks of the same sets of species could provide interesting insight into how interaction networks were built over evolutionary time (see also [44]). Finally, the combination of range overlap networks and phylogenetic comparative methods seems like a particularly promising area for future research to bridge local biotic interaction to large-scale phenotypic diversification, both in space and over time.

## Supporting information

S1 Fig Variation across nodes in simulated range overlap networks (with truncation), namely degree distribution, betweenness, and closeness. Top Row: simulations in which the standard deviation in the range size distribution was set to 1 on a log scale, while the mean of the range size distribution (meanlog) was varied systematically; Bottom Row: simulations in which the mean of the range size distribution was set to 0 on a log scale, while the standard deviation of the range size distribution (sdlog) varied systematically.
(EPS)

S2 Fig Comparison between empirical data on the altitudinal distribution of Neotropical birds and the expectations based on our model. The results showed are of simulations with truncation. Locations are: Madrian Highlands (MAH) (N = 384 species), Chiriquí-Darién Highlands (CDH) (N = 278 species), Central Andes (CAN) (N = 801 species), Northern Andes (NAN) (N = 768 species), and Tepuis (TEP) (N = 142 species): (A) altitudinal distribution of the species in each location, ranked according to the lowest limit to facilitate visualization; (B) altitudinal range size distribution; (C) altitudinal range overlap distribution (gray histogram indicates the expectation of the range overlap distribution based on 100 model simulations using mean and standard deviation calculated directly from the empirical data, and the colored and gray vertical lines indicate the observed and simulated mean range overlap, respectively); (D) distribution of the simulated number (n = 100) of overlapping species (histogram) and the observed means for each location (vertical lines); (E) degree distribution across nodes in the empirical datasets (colored lines) and expectations based on simulations (n = 50) (gray lines); (F) betweenness across nodes in the empirical datasets (colored lines) and expectations based on simulations (n = 50) (gray lines); (G) closeness across nodes in the empirical datasets (colored lines) and expectations based on simulations (n = 50) (gray lines).
(EPS)
